# Interaction Between CsATG8f and CsRAP2.12 Modulates Antioxidant Defense and Hypoxia Response During Submergence in *Camellia sinensis*

**DOI:** 10.3390/ijms27010235

**Published:** 2025-12-25

**Authors:** Rou Zeng, Yun Liu, Lisha Yu, Xiaogang Lei, Jie Jiang, Qiang Shen, Yuanchun Ma, Wanping Fang, Xujun Zhu

**Affiliations:** 1 College of Horticulture, Nanjing Agricultural University, Nanjing 210095, China; 2 Tea Research Institute, Guizhou Provincial Academy of Agricultural Sciences, Guiyang 417100, China

**Keywords:** submergence resistance, autophagy, ATG8, ROS scavenging, tea plants

## Abstract

Autophagy is an evolutionarily conserved cellular process that maintains homeostasis by degrading intracellular materials. Numerous studies have investigated the role of autophagy-related genes (ATGs) in plant adaptation to abiotic stresses. In plants, hypoxia (e.g., flooding events, oxygen supply during growth) rapidly activates the autophagy pathway as a protective mechanism for cell survival. Considering the moisture-loving yet waterlogging-sensitive nature of tea plants, this study explored the role of *CsATG8f* in the tea plant’s response to submergence. We found that overexpression of *CsATG8f* formed more autophagosomes than controls under submergence. Furthermore, CsATG8f was confirmed to physically interact with CsRAP2.12. Co-overexpression of both genes partially suppressed transcription of hypoxia-response genes while activating the antioxidant system, thereby enhancing tea plants’ resistance to submergence. Consistent with this, the opposite trend was observed in silenced plants, which attempted to mitigate stress damage by increasing GABA levels in vivo. In conclusion, our study reveals the crucial roles of *CsATG8f* and *CsRAP2.12* in tea plant tolerance to submergence and provides new insights into potential regulatory networks governing tea plant adaptation to flooding.

## 1. Introduction

Throughout their entire growth cycle, plants continuously encounter various environmental stresses that are unsuitable for growth and survival. These stresses include drought, flooding, salinity, and extreme weather conditions. Due to intensifying global climate change, drought and flooding now alternate with increased frequency, greater severity, and broader impact, leading to frequent and concurrent agricultural meteorological disasters [1]. Flooding stress has become one of the primary abiotic stresses in agricultural production, as most terrestrial plants can only tolerate submergence for short periods. This places plants in periodic or prolonged anaerobic or hypoxic states, restricting aerobic respiration and energy production essential for sustaining vital functions [2]. Flooding stress includes waterlogging and submergence. During waterlogging, restricted gas diffusion in and out of plant cells combined with continuous root respiration consuming environmental O_2_ rapidly induces hypoxia. This leads to impaired root functions such as reduced rooting rates and root rot, indirectly affecting aboveground plant functions. During submergence, beyond root impacts, dual constraints of low light and oxygen levels inhibit aboveground photosynthesis, reducing photosynthetic products and oxygen production. This causes severe shortages in plant energy metabolism and nutrient supply [3,4]. The resulting decrease in O_2_ and accumulation of CO_2_ alter cytoplasmic pH, disrupting cation-anion uptake balance, ATP synthesis becomes constrained, and reactive oxygen species accumulate extensively, ultimately severely disrupting normal physiological and biochemical processes [5,6,7,8,9]. Therefore, to cope with hypoxia stress caused by partial or complete submergence, plants have evolved various adaptive strategies. These include selecting ethanol fermentation over the high-yield mitochondrial oxidative phosphorylation pathway to generate glycolytic ATP, forming specialized structures (e.g., adventitious roots) that promote gas exchange, elongating internodes to access oxygen (escape strategy), and halting growth (quiescence strategy) to reduce energy expenditure [10,11,12,13].

Autophagy, or self-eating, is a highly conserved homeostatic regulatory recycling process for degradation in eukaryote organisms. During autophagy, cells form double-membrane vesicles called autophagosomes to engulf cellular contents. These are then transported to either the vacuole (in plants and yeast) or the lysosome (in animals). The outer membrane of the autophagosome fuses with the vacuolar or lysosomal membrane, exposing the inner membrane contents to the vacuolar or lysosomal lumen. where they are ultimately degraded by hydrolases [14]. Under normal conditions, such as leaf senescence and seed germination, autophagy occurs at low levels as a basal cleanup process, clearing unwanted cytoplasmic contents and reallocating nutrients for metabolic homeostasis. However, under certain stresses, autophagy is induced in plants to facilitate the recycling of damaged or unnecessary cellular material, thereby mitigating stress-induced damage [15]. Autophagy is currently classified into three types: macroautophagy, microautophagy, and chaperone-mediated autophagy. The primary distinction among them lies in the pathways through which cellular material is transported into the vacuole or lysosome. Research indicates that only macroautophagy and microautophagy exist in plants, with no chaperone-mediated autophagy. Macroautophagy is the predominant form (hereafter referred to as autophagy) and is activated during specific developmental stages or under various environmental stresses [16]. Previous studies revealed that inhibiting autophagy via 3-MA treatment or *ATG6* knockdown accelerates programmed cell death (PCD) in wheat seedlings under drought stress. Similarly, silenced *ATG2* or *ATG7* to suppress autophagy promotes PCD during salt stress [17,18]. Autophagy is also crucial for clearing reactive oxygen species (ROS) under anaerobic stress induced by submergence. Overexpression of apple autophagy-related genes *MdATG5a* and *MdATG8i* significantly alleviates oxidative damage under stress and activates autophagy to enhance plant stress resistance [19,20]. Waterlogging induces transcription of *Arabidopsis thaliana* (L.) hypoxia response genes and RBOH-mediated ROS production, while also increasing transcription levels of autophagy-related genes (ATGs) and autophagosome numbers [21]. Thus, autophagy plays a crucial role in plant resistance to environmental stress, and its activation is closely linked to the ATG family. Autophagy-related genes are essential for autophagosome formation, and phagosome generation requires the regulation of distinct autophagy-related proteins that are highly conserved across plants, yeast, and mammals [22]. Among numerous ATG proteins, ATG8 plays a central role in autophagosome formation, membrane fusion, and the recruitment of selective autophagy receptors (SARs) [23,24]. In selective autophagy, the interaction between ATG8 and selective autophagy receptors is crucial for precise cargo recognition. Receptors bind to ATG8 and ubiquitinate proteins, directing organelles and pathogens destined for degradation into autophagosomes. After autophagosome closure and fusion with the vacuole (or lysosome), the ATG8 protein on the inner membrane is degraded along with the cargo, while the remainder is recycled for reuse [25,26,27]. Consequently, the ATG8 protein is frequently used as an autophagosome marker to assess the induction level of autophagy.

The ethylene synthesis pathway is activated, and ethylene receptor gene expression is induced under hypoxia. The mechanism for sensing low O_2_ signals is closely linked to specific transcription factors, the ERF-VIIs (Ethylene Response Factor VII) [28,29,30]. Five groups of ERF-VIIs were first identified in *A. thaliana*: *HRE1* (*Hypoxia responsive 1*), *HRE2* (*Hypoxia responsive 2*), *RAP2.2* (*Related to AP2.2*), *RAP2.3* (*Related to AP2.3*), and *RAP2.12* (*Related to AP2.12*) [31,32]. Studies indicate that upregulating ERF-VII subfamily genes significantly enhances ADH1 enzyme activity and improves resistance to hypoxia stress [33,34]. ERF-VIIs in wheat and maize also participate in O_2_ sensing [35,36]. The ERF-VII transcription factor *RAP2.12* serves as a core molecular sensor for plants to perceive oxygen levels and trigger hypoxic (e.g., submergence) adaptive responses. It mediates plant perception and response to hypoxia stress through a highly conserved N-end rule pathway and subcellular localization regulation. Previous studies have elucidated the mechanism of direct O_2_ sensing in *A. thaliana*, where ERF-VII protein stability is regulated via the NERP (N-end rule pathway) degradation pathway to detect changes in intracellular oxygen levels [37]. Under normoxic conditions, RAP2.12 is anchored to the plasma membrane by binding to the ACBP1/2 (plasma membrane-localized acyl-CoA binding protein), preventing nuclear translocation. Upon encountering hypoxia, RAP2.12 released from the ACBP complex translocates to the nucleus, directly activating the expression of downstream hypoxia-response genes [38]. This systemically regulates plant energy metabolism balance and hypoxia stress adaptation capacity.

Plants activate complex signaling and metabolic networks as adaptive strategies when responding to submergence, with certain metabolites and bioactive molecules serving as key signaling molecules to mediate rapid stress responses. On one hand, the rapid accumulation of gamma-aminobutyric acid (GABA) is a significant characteristic. GABA is a four-carbon non-protein amino acid ubiquitous in both prokaryotes and eukaryotes. It is primarily metabolized via the GABA shunt pathway, bypassing the tricarboxylic acid (TCA) cycle, involving three enzymes: glutamate decarboxylase (GAD), GABA transaminase (GABA-T), and succinate semialdehyde dehydrogenase (SSADH) [39]. Under normal conditions, GABA levels in plant tissues are generally low but rise rapidly under hypoxia stress. Reports indicate an eightfold increase in GABA content in tea leaves under anaerobic. More intriguingly, GABA concentrations in tea seedlings further increased after alternating anaerobic and aerobic incubation [40,41]. In naked oats, both water immersion and vacuum treatment significantly elevated GABA content. Comparative studies revealed that low-oxygen environments were more conducive to GABA accumulation during germination than complete anoxia [42]. This accumulation enables GABA to function as a signaling molecule, mitigating stress damage through mechanisms including stomatal closure induction, ROS scavenging, ion balance regulation, and cellular homeostasis maintenance [43,44,45,46]. On the other hand, ROS, another crucial class of stress signaling molecules, also play a dual signaling role in submerged responses. ROS are byproducts of fundamental cellular activities in aerobic organisms, including hydrogen peroxide (H_2_O_2_), superoxide anion (O_2_^−^), and hydroxyl radical (OH^−^) [47,48]. Although ROS-mediated oxidative stress is generally considered toxic, low concentrations of ROS function as signaling molecules in stress responses [49,50]. Notably, stress-induced ROS accumulation can trigger autophagy, which in turn maintains ROS homeostasis by clearing damaged organelles, forming a finely tuned feedback regulatory loop that prevents oxidative damage [51].

Tea (*Camellia sinensis* (L.) Kuntze) is one of China’s most important economic crops, rich in nutrients such as theanine and catechins. It exhibits growth characteristics that favor warmth over cold and moisture over submergence. However, waterlogging caused by excessive soil moisture or impaired drainage inhibits normal tea plant growth, reduces tea yield and quality, and impairs the economic benefits of the tea industry. Research on the relationship between submergence and autophagy in tea plants remains limited. We reported the role of *CsATG8f* in inducing autophagy during submergence in tea plants, verified its interaction with *CsRAP2.12*, and demonstrated that both are essential for regulating the plant’s response to hypoxia under submergence.

## 2. Results

### 2.1. Expression Pattern of CsATG8f

**Figure 1:**
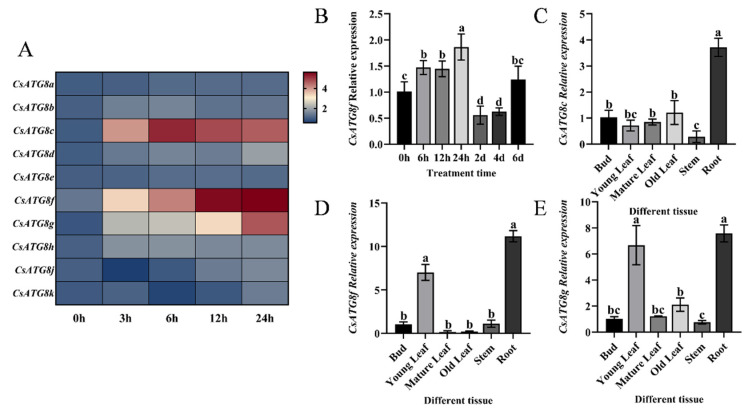
Expression profile of *CsATG8f*. (**A**) Expression patterns of the CsATG8 gene family under vacuum treatment. (**B**) Expression characteristics of *CsATG8f* under submergence pretreatment. (**C**) Differential expression of *CsATG8c* in different tissues of tea plants. (**D**) Differential expression of *CsATG8f* in different tissues of tea plants. (**E**) Differential expression of *CsATG8g* in different tissues of tea plants. Data are presented as mean ± SD (*n* = 3). Different letters indicate statistically significant differences among groups (*p* < 0.05, one-way ANOVA followed by Duncan’s multiple range test).

Initially, vacuum-induced hypoxia stress was employed to observe the expression patterns of *CsATG8s* in tea plants. Quantitative reverse transcription-polymerase chain reaction (qRT-PCR) was then used to investigate the expression dynamics of *CsATG8s* at different time points under this treatment. Most genes exhibited varying degrees of induction under hypoxia stress relative to their respective controls. Among them, *CsATG8c*, *CsATG8f*, and *CsATG8g* showed relatively significant changes to hypoxia stress in tea plants (Figure 1A). The expression patterns of *CsATG8c*, *CsATG8f*, and *CsATG8g* varied across different tissues: *CsATG8c* showed the highest expression in roots (Figure 1C), while *CsATG8f* was expressed in young leaves and roots with significantly higher levels than other tissues (Figure 1D). A similar pattern was observed for *CsATG8g* (Figure 1E).

Given that tea plants’ economic value is primarily determined by their leaves, and *CsATG8f* expression levels continuously increased with prolonged hypoxia stress duration. *CsATG8f* was selected for subsequent studies to elucidate the molecular mechanisms underlying tea plants’ response to submergence. Tea plants were subjected to complete submergence to simulate the hypoxic environment induced by waterlogging. qRT-PCR analysis of *CsATG8f* expression patterns under this treatment revealed a responsive pattern, with expression levels peaking at 24 h post-submergence (Figure 1B).

### 2.2. Cloning and Functional Characterization of the CsATG8f Gene

**Figure 2:**
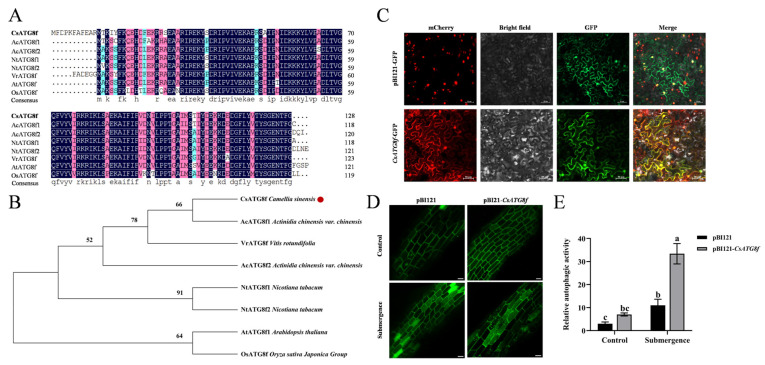
Sequence information and functional analysis of *CsATG8f*. (**A**) Protein domain alignment of ATG8f from different species. (**B**) Phylogenetic analysis of ATG8f from different species. (**C**) Subcellular localization of *CsATG8f*. (**D**) Number of autophagosomes in tea plant roots under different treatments. (**E**) Visualization of autophagic activity in tea plant roots. In (**C**,**D**), Scale bar = 50 µm. In (**E**), data are presented as mean ± SD (*n* = 8). Different letters indicate statistically significant differences among groups (*p* < 0.05, one-way ANOVA followed by Duncan’s multiple range test).

To analyze the sequence characteristics of *CsATG8f*, a 384 bp open reading frame (ORF) was cloned from ‘Zhongcha 108’ using homologous cloning, and the protein encoded by the *CsATG8f* gene was analyzed. We compared its amino acid sequence with those from other species. Similarly to its homologs in *A. thaliana*, Muscadine (*Vitis rotundifolia* Michx.), tobacco (*Nicotiana tabacum* L.), Chinese kiwi (*Actinidia chinensis* Planch. var. *chinensis*), and rice (*Oryza sativa* L. subsp. *japonica*), the tea plant CsATG8f contains a highly conserved Ub1_ATG8 domain. The sequences of the Ubl_ATG8 domain across these eight proteins showed minor differences, with 86 identical amino acids (Figure 2A). Subsequently, a phylogenetic tree was constructed using MEGA 11 software to compare their evolutionary relationships. The amino acid sequence encoded by *CsATG8f* is most closely related to *A. chinensis* var. *chinensis*, followed by *V. rotundifolia* (Figure 2B).

To investigate the function of *CsATG8f* in tea plants, multiple subcellular localization prediction websites indicated that the CsATG8f protein localizes to the nucleus and cytoplasm. To validate this prediction, the *CsATG8f* coding sequence (CDS) was fused in-frame with the gene encoding green fluorescent protein (GFP). The resulting construct was introduced into tobacco epidermal tissues via Agrobacterium-mediated transformation, alongside a nucleocytoplasmic marker for co-expression. The results were observed under a confocal microscope. In the control empty vector pBI121-GFP, green fluorescence signals were detected in the cytoplasm, cell membrane, and nucleus. However, the expression vector containing the *CsATG8f* gene fragment showed green fluorescence signals exclusively in the nucleus and cytoplasm, overlapping with the marker (Figure 2C). This confirms that *CsATG8f* localizes to the nucleus and cytoplasm, consistent with the prediction.

Next, to verify whether *CsATG8f* expression responds to submergence and autophagosome production in tea plant. We observed changes in autophagosomes in *CsATG8f* transiently overexpressed tea plant roots and visualized them to determine autophagic activity. As shown in Figure 2D,E, under normal conditions, both control and *CsATG8f*-overexpression plant roots exhibited few autophagosomes. Following submergence treatment, autophagosome numbers increased in all root systems. Notably, *CsATG8f*-overexpression plant roots displayed significantly more autophagosomes than control.

### 2.3. CsATG8f Interacts with CsRAP2.12

To elucidate the molecular mechanism by which *CsATG8f* regulates tea plant tolerance to submergence, we identified potential interacting proteins of *CsATG8f* using a yeast two-hybrid screening approach. Prior to the screening, the toxicity and self-activation of the bait vector pGBKT7-*CsATG8f* in yeast were assessed. Using the PclI plasmid as a positive control and the pGBKT7 plasmid as a negative control, we observed that only the positive control group grew normal yeast colonies and turned blue on SD/-His/-Ade/X-α-gal deficient medium. while neither the negative control nor the bait construct pGBKT7-*CsATG8f* produced blue yeast colonies (Figure S1). This indicates that *CsATG8f* does not exhibit autoactivation in yeast, allowing subsequent experiments to proceed. Further validation through yeast two-hybrid screening, combined with colony PCR and sequencing, revealed that *CsATG8f* likely interacts with *CsRAP2.12*, a transcription factor responsive to hypoxia. To exclude potential false positives in the yeast two-hybrid screening, one-to-one yeast two-hybrid reciprocal validation and additional verification methods were required to confirm the interaction. Therefore, we also performed firefly luciferase complementation imaging (LCI) assay and bimolecular fluorescence complementation (BiFC) assay in tobacco leaves.

**Figure 3:**
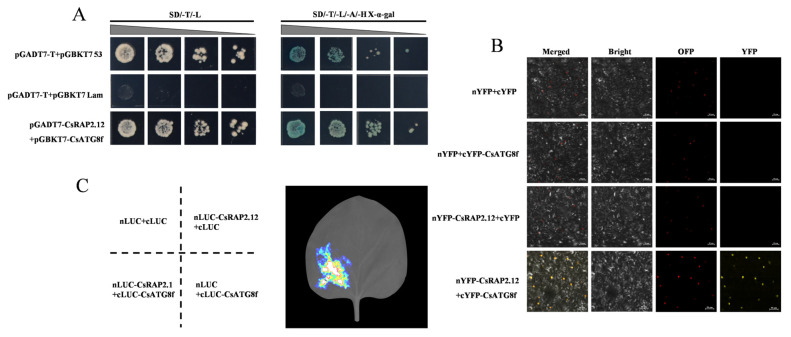
CsATG8f and CsRAP2.12 exhibit physically interacts. (**A**) Y2H assay shows CsATG8f and CsRAP2.12 co-transformed cells growing on selective medium (SD/-T/-L/-A/-H + X-α-gal) and displaying blue coloration. (**B**) BiFC assay demonstrates the interaction between CsATG8f and CsRAP2.12 in tobacco leaf epidermal cells. Scale bar = 50 µm. (**C**) LCI assay confirms the interaction between CsATG8f and CsRAP2.12 in tobacco leaves.

The full-length CDS of this transcription factor was cloned from ‘Zhongcha 108’ and recombined into the pGADT7 vector to obtain the pGADT7-*CsRAP2.12* construct for yeast two-hybrid reciprocal validation. Yeast two-hybrid one-on-one validation revealed that both the positive control pGADT7-T + pGBKT7-53 and the experimental group pGADT7-*CsRAP2.12* + pGBKT7-*CsATG8f* grew normally and exhibited blue coloration on SD/-Ade/-His/-Leu/-Trp/X-α-gal deficient medium. while the negative control pGADT7-T + pGBKT7-Lam failed to form yeast colonies, confirming CsRAP2.12 protein interacts with CsATG8f in yeast cells (Figure 3A). Similar results were obtained in BiFC assay and LCI assay. LCI analysis revealed luciferase emission upon co-infiltration of nLUC-*CsRAP2.12* and cLUC-*CsATG8f* in leaf tissue (Figure 3C), indicating strong interaction between CsATG8f and CsRAP2.12 in plant leaf cells. In the BiFC assay, strong YFP fluorescence signals were detected in tobacco leaf cell nuclei when co-infiltrating nYFP-*CsRAP2.12* and cYFP-*CsATG8f*, whereas no YFP fluorescence was observed in any negative control group (Figure 3B), further confirming the interaction between CsATG8f and CsRAP2.12 in vivo. Collectively, these results indicate that a physical interaction occurs between the CsATG8f and CsRAP2.12 proteins.

To investigate whether the function of *CsRAP2.12* is conserved in tea plants, sequence analysis revealed that *CsRAP2.12* belongs to the AP2/ERF gene family and possesses a typical AP2 conserved domain (Figure S2A). It exhibits high similarity to homologs in *A. chinensis* var. *chinensis*, *N. tabacum*, *Populus trichocarpa* (Torr. & Gray), *A. thaliana*, grape (*Vitis vinifera* L.), and tomato (*Solanum lycopersicum* L.). Phylogenetic analysis revealed that *CsRAP2.12* is most closely related to *A. chinensis* var. *chinensis*, followed by *V. vinifera* (Figure S2B).

### 2.4. Assay of Transient Overexpression of CsATG8f and CsRAP2.12 Under Submergence

**Figure 4:**
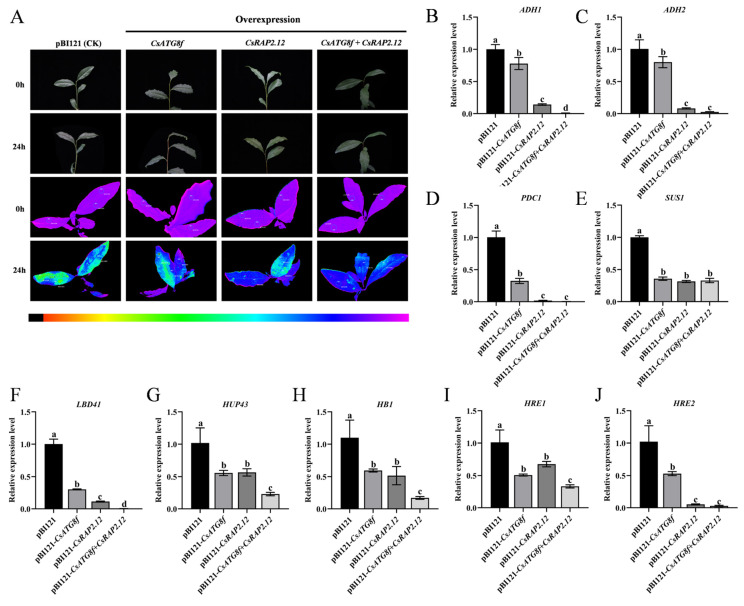
Expression changes in stress-related genes following transient overexpression of *CsATG8f* and *CsRAP2.12*. (**A**) Phenotypic and in vivo chlorophyll fluorescence imaging before and after 24 h of submergence. (**B**–**E**) Expression changes in fermentation-related genes. (**F**–**J**) Expression changes in hypoxia-response genes. Data are presented as mean ± SD (*n* = 3). Different letters indicate statistically significant differences among groups (*p* < 0.05, one-way ANOVA followed by Duncan’s multiple range test).

To investigate the potential roles of *CsATG8f* and *CsRAP2.12* in tea plant tolerance to submergence, Agrobacterium-mediated transformation was used to inject tea leaves with bacterial suspension containing expression vectors carrying target gene fragments, thereby upregulating the expression levels of *CsATG8f* and *CsRAP2.12* in tea plants. The transient overexpression effects of the *CsATG8f* and *CsRAP2.12* genes are shown in Supplementary Material (Figure S3A). In the Fv/Fm pseudo-color image, pure purple indicates normal photosynthetic system status, while blue and green indicate damage to photosystem II caused by stress treatments. Consequently, under combined submergence and transient overexpression of target genes, lines transiently overexpression target genes exhibited weaker green fluorescence than the control, with the weakest green fluorescence observed in co-transiently overexpressed plant pBI121-*CsATG8f* + *CsRAP2.12* (Figure 4A). This indicates that the overexpressed lines suffered less stress damage than the control, and the reduction in damage was most pronounced when *CsATG8f* and *CsRAP2.12* were co-transiently overexpressed. Simultaneously, qRT-PCR was employed to detect the expression of hypoxia marker genes following submergence: *alcohol dehydrogenase* (*ADH1* and *ADH2*), *pyruvate decarboxylase 1* (*PDC1*), and *sucrose synthase 1* (*SUS1*), as well as hypoxia-response genes, including *hypoxia-response unknown protein 43* (*HUP43*), *hemoglobin 1* (*HB1*), and *lob domain containing protein 41* (*LDB41*). Transcript levels of the ethylene-responsive *hypoxia-response ERF* (*HRE1* and *HRE2*) were also measured, as ethylene is widely recognized as a key regulator of plant hypoxia signaling. qRT-PCR analysis revealed that compared to the control, hypoxia-stress-related gene transcripts in overexpression lines were significantly upregulated after submergence. Particularly in co-transiently overexpressed plant pBI121-*CsATG8f* + *CsRAP2.12*, the expression levels of some hypoxia-stress-related genes in this overexpression lines were lower, except for *ADH2*, *PDC1*, *SUS1*, and *HRE2* (Figure 4B–J). Collectively, these results indicate that transient overexpression of either *CsATG8f* or *CsRAP2.12* individually confers a less sensitive phenotype to submergence compared to the control. This insensitive phenotype was most pronounced when *CsATG8f* and *CsRAP2.12* were co-transiently overexpressed.

### 2.5. Assay of Transient Silenced of CsATG8f and CsRAP2.12 Under Submergence

**Figure 5:**
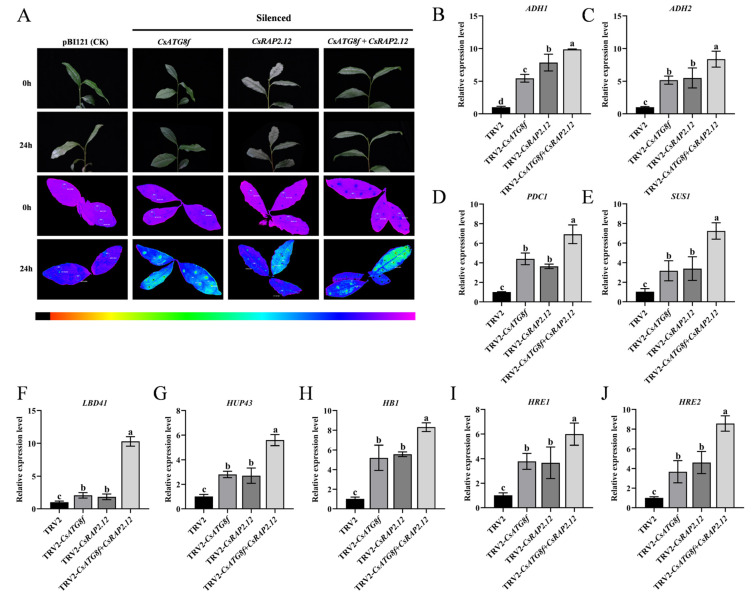
Expression changes in stress-related genes after transient silencing of *CsATG8f* and *CsRAP2.12*. (**A**) Phenotypic and in vivo chlorophyll fluorescence imaging before and after 24 h of submergence. (**B**–**E**) Expression changes in fermentation-related genes. (**F**–**J**) Expression changes in hypoxia-response genes. Data are presented as mean ± SD (*n* = 3). Different letters indicate statistically significant differences among groups (*p* < 0.05, one-way ANOVA followed by Duncan’s multiple range test).

To better elucidate the roles of *CsATG8f* and *CsRAP2.12* in submergence, virus-induced gene silenced (VIGS) was employed to silence the *CsATG8f* and *CsRAP2.12* genes in tea plants. The transient silenced effects of the *CsATG8f* and *CsRAP2.12* genes are shown in Supplementary Material (Figure S3B). Compared to control, TRV-*csatg8f* and TRV-*csrap2.12* plants exhibited brighter green fluorescence. Notably, TRV-*csatg8f* + *csrap2.12* plants displayed even more intense fluorescence than TRV-*csatg8f* and TRV-*csrap2.12* plants (Figure 5A). Similarly, the expression levels of hypoxia marker genes and hypoxia-response genes in silenced lines after submergence were examined, as shown in Figure 5B–J. The transcripts of hypoxia-related genes in silenced lines were significantly up-regulated after submergence. Notably, when *CsATG8f* and *CsRAP2.12* were transiently silenced together, the increase in expression exceeded that observed in either TRV-*CsATG8f* or TRV-*CsRAP2.12* plants alone. Collectively, these results indicate that both TRV-*csatg8f* and TRV-*csrap2.12* are more sensitive to submergence than the control, with the TRV-*csatg8f* + *csrap2.12* exhibiting the most severe sensitivity phenotype.

### 2.6. Effects of Hypoxia Stress Induced by Submergence on ROS Accumulation and Antioxidant System in Tea Plants

**Figure 6:**
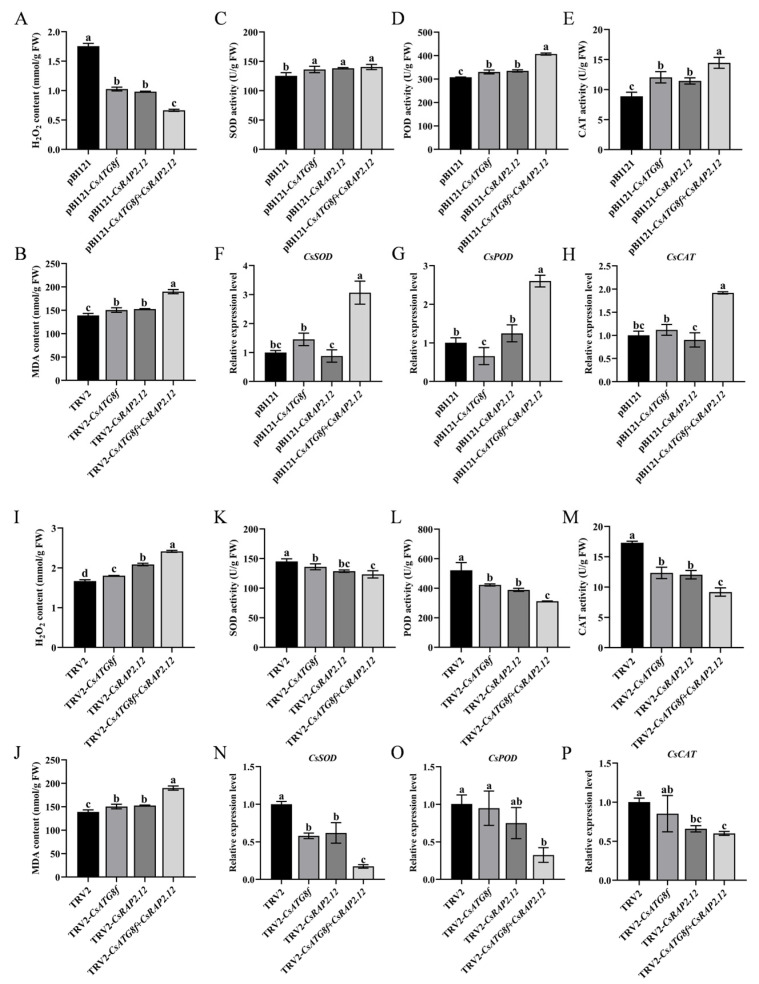
Effects of *CsATG8f* and *CsRAP2.12* on the oxidative stress system of tea plants under submergence. (**A**–**H**) Transient overexpression of target genes: (**A**) H_2_O_2_ content after 24 h of submergence, (**B**) MDA content after 24 h of submergence, (**C**–**E**) SOD/POD/CAT activity after 24 h of submergence, (**F**–**H**) *CsSOD*/*CsPOD*/*CsCAT* transcript levels after 24 h of submergence. (**I**–**P**) Transient silencing of target genes: (**I**) H_2_O_2_ content after 24 h of submergence, (**J**) MDA content after 24h of submergence, (**K**–**M**) SOD/POD/CAT activity after 24 h of submergence, (**N**–**P**) *CsSOD*/*CsPOD*/*CsCAT* transcript levels after 24 h of submergence. Data are expressed as mean ± SD (*n* = 3). Different letters indicate statistically significant differences among groups (*p* < 0.05, one-way ANOVA followed by Duncan’s multiple range test).

The tolerance of plants to abiotic stress is closely related to the capacity of their antioxidant system to scavenge ROS. To determine the antioxidant capacity of tea plants after submergence, we measured hydrogen peroxide (H_2_O_2_) content, malondialdehyde (MDA) content, and the activity of antioxidant enzymes (SOD, POD, and CAT) in transiently overexpressed and transiently silenced plants, respectively. We observed that, in the transient overexpression lines, H_2_O_2_ and MDA levels were significantly reduced compared to control (Figure 6A,B), while superoxide dismutase (SOD), peroxidase (POD), and catalase (CAT) activities were markedly enhanced—particularly when *CsATG8f* and *CsRAP2.12* were co-transiently overexpressed (Figure 6C–E). In silenced lines, SOD, POD, and CAT activities in TRV-*csatg8f* and TRV-*csrap2.12* plants were significantly reduced compared to control, with TRV-*csatg8f* + *csrap2.12* plants exhibiting the lowest activity (Figure 6K–M). In contrast, H_2_O_2_ and MDA contents were significantly increased in all silenced lines compared to control, with the most pronounced increase observed in TRV-*csatg8f* + *csrap2.12* plants (Figure 6A,B). Next, we examined the gene expression levels of antioxidant enzymes (SOD, POD, and CAT). The results showed that the trends in transcript levels were similar to those observed in enzyme activity. In the overexpression lines, *CsSOD*, *CsPOD*, and *CsCAT* transcript levels were highest in the co-overexpression plant pBI121-*CsATG8f* + *CsRAP2.12*, significantly exceeding those in the single-overexpression plants pBI121-*CsATG8f* and pBI121-*CsRAP2.12*, as well as the control (Figure 6F–H). Conversely, in the silenced lines, *CsSOD*, *CsPOD*, and *CsCAT* transcript levels in the co-silenced plant TRV-*csatg8f* + *csrap2.12* were significantly lower than those in the single-silenced plants TRV-*csatg8f* and TRV-*csrap2.12*, as well as the control (Figure 6N–P).

### 2.7. Effects of Hypoxia Stress Induced by Submergence on Endogenous GABA in Tea Plants

**Figure 7:**
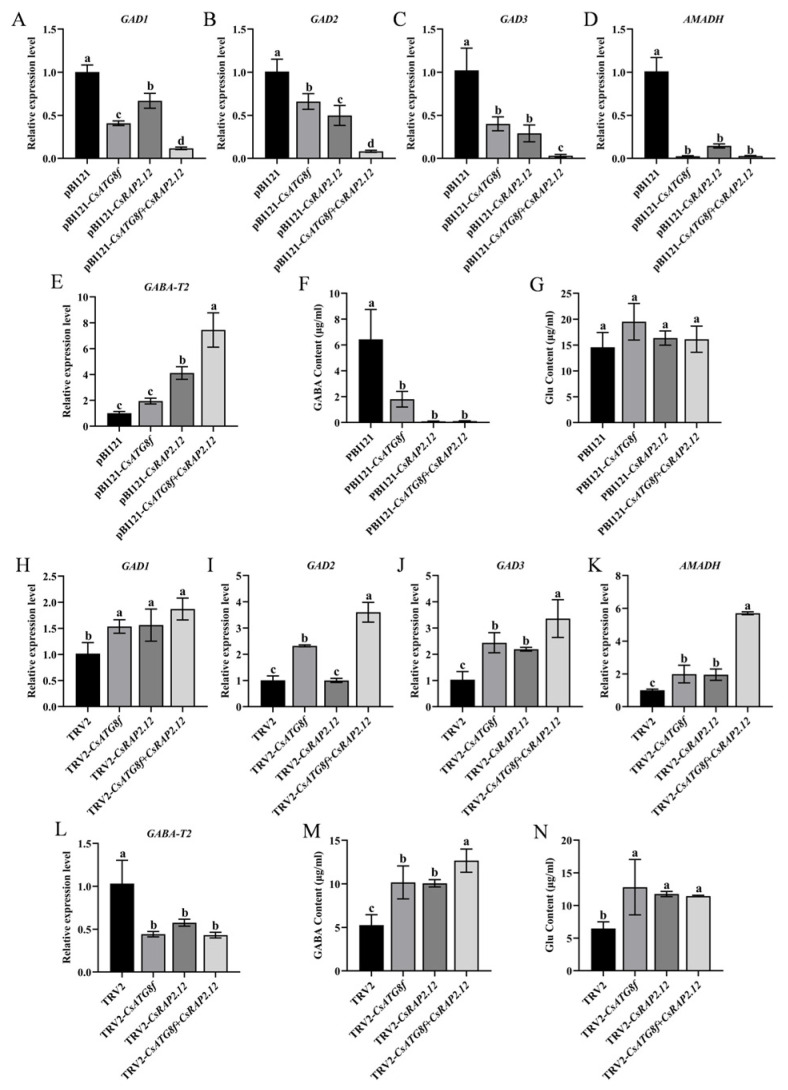
Effects of *CsATG8f* and *CsRAP2.12* on endogenous GABA levels in tea plants under submergence. (**A**–**G**) Transient overexpression of target genes: (**A**–**E**) Transcript levels of genes related to GABA synthesis and degradation after 24 h submergence; (**F**) Glutamate (Glu) content after 24 h of submergence; (**G**) GABA content after 24 h of submergence. (**H**–**N**) Transient silencing of target genes: (**H**–**L**) Transcript levels of genes related to GABA synthesis and degradation after 24 h of submergence; (**M**) Glutamate (Glu) content after 24 h of submergence; (**N**) GABA content after 24 h of submergence. Data are presented as mean ± SD (*n* = 3). Different letters indicate statistically significant differences among groups (*p* < 0.05, one-way ANOVA followed by Duncan’s multiple range test).

As described in the introduction, GABA functions as a signaling molecule involved in regulating tolerance to various abiotic stresses. To investigate whether tea plants induce GABA accumulation to enhance tolerance under hypoxia, we measured glutamate (Glu) and GABA contents in tea plants subjected to transient overexpression or transient silenced. GABA levels were significantly lower than the control in transient overexpression lines, while Glu content—the precursor for GABA synthesis—showed no significant difference from the control (Figure 7F,G). To further investigate the effects of transiently overexpressed target gene on the GABA shunt, qRT-PCR analysis was performed on *Glu dehydrogenase* (*GAD1*, *GAD2*, and *GAD3*), *GABA transferases* (*GABA-T2*), and *succinate semialdehyde dehydrogenase* (*AMADH*) gene expression. The results revealed that the overexpression lines exhibited significantly lower expression levels of GABA synthesis-related genes (*GAD1/2/3* and *AMADH*) under submergence compared to the control, while *GABA-T2* expression were significantly increased (Figure 7A–E). These results indicate that, unlike the overexpression lines, the control group attempted to accumulate more GABA under submergence to resist stress-induced damage. Interestingly, the opposite result was observed in transient silenced lines. GABA content in silenced lines was significantly higher than in the control, particularly in TRV-*csatg8f* + *csrap2.12* plants. Similarly, Glu content was significantly increased compared to the control (Figure 7M,N). qRT-PCR analysis of GABA shunt gene expression revealed that silenced lines exhibited significantly higher expression levels of GABA synthesis-related genes under submergence compared to the control, while *GABA-T2* expression levels were significantly reduced (Figure 7H–L). This indicates that after silenced *CsATG8f* and *CsRAP2.12*, tea plants experienced more severe submergence than the control, thereby promoting increased GABA synthesis to counteract the stress.

## 3. Discussion

### 3.1. CsATG8f Enhances Tea Plant Tolerance to Submergence by Promoting Autophagy

When plants encounter environmental stress, autophagy—a highly conserved degradation pathway—plays a crucial role in helping plants regulate metabolism and repair damage. In tomatoes, HsfA1a induces autophagy to degrade insoluble proteins produced under drought stress, thereby enhancing tomato drought tolerance [52]. *A. thaliana atg5* and *atg10* mutants exhibit accelerated senescence under zinc deficiency conditions [53]. Overexpression of *MdATG18a* increases autophagy activity and improves apple tolerance to nitrogen starvation [54]. In *A. thaliana*, overexpression of two members of the tea plant CsATG3 gene family, *CsATG3a* and *CsATG3b*, yielded consistent results [55]. Thus, autophagy acts as a protective mechanism throughout the plant growth cycle. The ATG8 gene family encodes ubiquitin-like proteins. The ATG8-PE conjugation pathway, involving the lipid phosphatidylethanolamine, not only regulates autophagosome membrane expansion and closure but is also present throughout the autophagy process, being essential for autophagosome formation [56,57,58]. The function of the plant ATG8 gene family under abiotic stress has been extensively reported. During heat stress, ATG8 co-immunoprecipitates with diverse heat shock proteins (HSP90s, HSP101, and HSP17.6), and their accumulation occurs in autophagy-deficient conditions [59]. Overexpression of *AtATG8* enhances autophagy flux, improving *A. thaliana* tolerance to salt and osmotic stress [60]. S-NITROSOGLUTATHIONE REDUCTASE1 (AtGSNOR1) interacts with AtATG8 to regulate seed germination under hypoxic conditions via selective autophagy [61]. Overexpression of *MaATG8f* enhances drought tolerance in bananas by modulating reactive oxygen species metabolism, abscisic acid biosynthesis, and increasing autophagy activity [62]. Although the ATG family has been identified in tea plants, current research on autophagy stress responses mediated by ATGs in tea has primarily focused on nutrient starvation stress [63,64]. Little is known about autophagy in tea plants responding to hypoxia induced by submergence, and its regulatory mechanisms remain unclear. In this study, we observed increased autophagosome formation in all root tissues following submergence. Furthermore, pBI121-*CsATG8f* plant exhibited significantly higher autophagosome numbers in roots compared to control (Figure 2D,E). This indicates that *CsATG8f* overexpression promotes autophagy in tea roots under submergence. This finding parallels previous research where overexpression of *MdATG10* enhanced cadmium stress tolerance in apple by increasing autophagic activity [65]. Phylogenetic tree and conserved domain analysis revealed that the function of *ATG8f* is conserved across these species despite evolutionary divergence (Figure 2A,B). Furthermore, *CsATG8f* was observed to localize to both the nucleus and cytoplasm (Figure 2C), suggesting a functional association with its gene. Under non-stress conditions, ATG8 proteins primarily reside in the cytoplasm. However, during adverse environmental conditions or specific stages of plant development, ATG8 proteins are recruited to different locations to perform their functions [66]. The C1 nucleoprotein (a viral replication-essential protein) of Tomato leaf curl Yunnan virus (TLCYnV) interacts with ATG8h, leading to its translocation from the nucleus to the cytoplasm and reduced accumulation of its own protein [67]. The colocalization of transcription factor *Dwg* with *ATG8a* is influenced by Drosophila starvation status: under feeding conditions, they colocalize in the nucleus, whereas under starvation conditions, they colocalize in the cytoplasm [68]. Therefore, we speculate that the cellular localization of *CsATG8f* is closely linked to its role in autophagy formation and function.

### 3.2. CsATG8f Interacts with CsRAP2.12 to Positively Regulate Tea Plant Tolerance to Submergence

*CsRAP2.12* is a member of the ERF VII subfamily and possesses the AP2 domain characteristic of the AP2/ERF family (Figure S2A). AP2/ERF transcription factors (TFs) not only serve as terminal response genes in the ethylene signaling pathway but also feedback-regulate plant hormone biosynthesis, acting as key regulators linking autophagy induction, plant hormone signaling, and stress responses. Transcriptome analyses under various stresses reveal significant changes in the expression of autophagy-related genes and ethylene response factors (ERFs) [69,70,71]. ERF genes, particularly members of Clusters II and VII, demonstrate significant potential for enhancing plant stress resistance. Overexpression of these genes improves transgenic plant tolerance to various biotic stresses (e.g., pathogen infection) and abiotic stresses (e.g., drought, salinity, waterlogging) [72]. Among these, ERF-VIIs have emerged as crucial molecular hubs regulating plant hypoxia sensing and signaling. In grape, VvERF-VIIs activate anaerobic metabolism and autophagy-mediated macromolecular catabolism, functioning as part of the energy regeneration mechanisms [73]. The maize *ZmEREB179* gene has been identified as a negative regulator of waterlogging stress, targeting the promoter region of *ZmEREB180* to suppress its expression [74]. This reveals a transcriptional cascade involving ERF-VIIs genes in regulating plant waterlogging tolerance. The wheat *BERF1* gene, encoding the protein most closely related to *A. thaliana AtRAP2.12* in sequence, functions as a hypoxic homeostasis sensor via the N-degron pathway [75]. The ACBP4-WRKY70 module integrates lipid metabolism (oleoyl-CoA) and phosphorylation signaling by regulating *RAP2.12* transcription, forming a dynamic response network that enables rapid plant adaptation to hypoxia stress [76]. Although extensive studies indicate that autophagy and *RAP2.12* mediated hypoxic response pathways both play crucial roles in submergence, their synergistic regulatory relationship remains unclear. In this study, we confirmed the physical interaction between the autophagy gene *CsATG8f* and the hypoxia-response transcription factor *CsRAP2.12* (Figure 3A–C). This finding may facilitate further understanding of the synergistic interaction between autophagy and hypoxia response factors during submergence adaptation. In functional validation assay, the overexpression lines exhibited reduced injury severity compared to control after submergence (Figure 4A). Surprisingly, multiple hypoxia-response genes and key anaerobic metabolism genes, including *ADH1*, *HUP43*, *LBD41*, and *HB1*, showed down-regulated expression in the overexpression lines. Notably, pBI121-*CsATG8f* + *CsRAP2.12* exhibited more pronounced downregulation than other groups (Figure 4B–J). Conversely, gene-silenced experiments yielded opposite results. The silenced lines exhibited increased damage severity compared to the control after submergence (Figure 5A). Concurrently, the expression levels of some hypoxia-response genes and key anaerobic metabolism genes were significantly up-regulated in the silenced lines. Notably, the TRV-*csatg8f* + *csrap2.12* plant showed a more pronounced upregulation than other groups (Figure 5B–J). Based on these findings, we conclude that *CsATG8f* and *CsRAP2.12* act as positive regulators of tea plant adaptation to submergence. The interaction between CsATG8f and CsRAP2.12 may synergistically enhance this positive regulatory relationship.

Notably, our findings that silenced *CsATG8f* resulted in an upward trend in expression levels of downstream hypoxia-response genes following submergence differ from previous reports. In *A. thaliana atg* mutants, elevated expression of *ADH1* and *PDC1* was observed after complete submergence, while expression levels of other hypoxia- and ethylene-responsive genes decreased [77]. This discrepancy may stem from differences in submergence duration and experimental materials. An earlier study on hypoxia response specificity revealed that several oxidative stress-related genes and hypoxia-inducible transcription factors in rice were suppressed or unchanged under hypoxia, exhibiting results opposite to those in *A. thaliana* [78,79]. Compared to *Avicennia marina*, *Aegiceras corniculatum* relies more heavily on *ADH* and *LDH* expression and adapts by downregulating *XET*, *SAMS*, and *ACCO* genes involved in cell wall and ethylene production [80]. Additionally, *RAP2.12* activity is regulated by the hypoxia-inducible transcription repressor *HRA1*. During early hypoxia, stable *RAP2.12* expression promotes transcription of downstream response genes, enhancing tolerance to low-oxygen stress. Under prolonged hypoxia, HRA1 protein binds to RAP2.12, inhibiting its activity and limiting the intensity and duration of the hypoxia response [81,82]. This feedback pathway indicates that under prolonged hypoxia, transcription of certain hypoxia response genes is downregulated to prevent plant energy depletion. This is not the case during the early stages. In *Taxodium hybrid* ‘Zhongshanshan 406’, *ThADH1* and *ThADH4* gene expression increased after complete submergence and persisted until day 50, while in soybean, *GmADH2* gene expression peaked within 6 h after flood stress [83,84]. This indicates that different plant species exhibit varying response times to submergence. These differences also suggest divergent regulatory strategies among plant species in responding to submergence. Furthermore, our study found that overexpression of *CsRAP2.12* did not enhance the expression of downstream hypoxia-response genes, and silencing it yielded the opposite result. Given that *RAP2.12* regulates transcription of downstream hypoxia-response genes while being negatively feedback-regulated by *HRA1*, we speculate that *CsRAP2.12* overexpression in tea plants stimulated the repressor *HRA1*, thereby enhancing its transcriptional inhibition of *CsRAP2.12* and reducing the transcriptional levels of response genes such as *ADH1*, *PDC1*, and *HB1*. Conversely, silenced *CsRAP2.12* in tea plants weakened *HRA1*’s inhibitory effect, allowing downstream hypoxia-response genes to rapidly increase their transcription levels upon submergence. The expression of hypoxia-response genes may reflect cellular “compensatory efforts” or a “feedback inhibition state”. While *HRA1*’s negative feedback is a possible mechanism, changes in hypoxia-response gene transcription levels may also stem from metabolic reprogramming or other unknown regulatory pathways. Furthermore, the negative correlation between upregulation of tea plant hypoxia-response genes and sensitive phenotypes may result from decreased post-translational efficiency. Previous studies revealed that plants under hypoxia readily accumulate excessive ROS, leading to oxidative damage, insufficient ATP supply, or even energy collapse, which suppresses global translation [85,86]. Reduced ATP content also diminishes the activity of the energy sensor TOR, thereby weakening ERF-VII protein-induced hypoxia response genes [87].

### 3.3. CsATG8f Interacts with CsRAP2.12 to Enhance the Antioxidant System and Improve Tea Plant Tolerance to Submergence

Under hypoxic conditions, constitutively expressed ERF-VII transcription factors redundantly regulate *A. thaliana* hypoxic adaptation [34,88], while autophagy-deficient mutants *atg4a/4b*, *atg5*, and *atg7* exhibit sensitivity to waterlogging [89]. Collectively, these findings indicate that autophagy and ERF-VII transcription factors play crucial roles in responding to submergence. Further analysis of our interaction and functional validation results revealed an interesting phenomenon: pBI121-*CsATG8f* + *CsRAP2.12* maintained superior submergence tolerance despite down-regulated expression of hypoxia-response genes, suggesting the potential existence of alternative regulatory mechanisms independent of the canonical fermentation pathway. This mechanism may involve multi-level regulation, such as maintaining protein quality control, energy metabolism balance, or redox homeostasis. Reduced plant stress tolerance is often associated with increased accumulation of insoluble ubiquitinated proteins, while autophagy maintains cellular homeostasis by clearing and recycling misfolded or denatured proteins [90,91,92]. For example, the C53/UFL1/DDRGK1 receptor complex maintains protein homeostasis under endoplasmic reticulum protein toxicity stress caused by ribosomal stalling through selective autophagy [93]. Regarding energy metabolism, submergence forces plants to shift toward low-yield pathways such as glycolysis and anaerobic fermentation, favoring the less energy-consuming sucrose synthase-catalyzed pathway [29,94]. Accumulation of ethanol, the end product of anaerobic fermentation, has been shown to enhance autophagy-mediated submergence tolerance [95]. In *A. thaliana*, the IQD22 protein was found to coordinate calcium-dependent activation of anaerobic fermentation within the CPK12-RAP2.12 and CaM-ADH1 regulatory modules, thereby controlling metabolic flux during hypoxia [96]. Autophagy defects readily lead to overall amino acid depletion and secondary metabolite accumulation, consistent with catabolic reprogramming and altered energy conversion triggered by impaired protein degradation [97]. Additionally, autophagy and ERF-VII transcription factors both play central roles in mitigating plant oxidative stress and coordinating redox signaling. ERF-VIIs exert their effects by promoting intracellular nitric oxide (NO) clearance and suppressing the expression of oxidative stress-related genes [98,99,100]. Autophagy, meanwhile, serves as a key mechanism for ROS clearance and ion homeostasis regulation during submergence stress [101,102]. The interaction between CsATG8f and CsRAP2.12 likely occupies a central position within these regulatory networks, though the precise details remain to be elucidated.

To investigate its mechanism, we examined whether the interaction between CsATG8f and CsRAP2.12 affects the antioxidant system of tea plants after submergence by measuring the antioxidant capacity of overexpression and silenced lines. Results revealed that overexpression of either pBI121-*CsATG8f* or pBI121-*CsRAP2.12* significantly enhanced SOD, CAT, and POD activities under submergence. Notably, pBI121-*CsATG8f* + *CsRAP2.12* demonstrated markedly higher ROS-scavenging enzyme activity compared to other groups (Figure 6C–E). Consistent with this, H_2_O_2_ accumulation was significantly reduced in these lines compared to the control, and MDA content was markedly decreased (Figure 6A,B). The silenced lines exhibited the opposite trend. Compared to the control, H_2_O_2_ accumulation was significantly increased, antioxidant enzyme activity was markedly reduced, and membrane lipid peroxidation was exacerbated in the silenced lines, with TRV-*csatg8f* + *csrap2.12* showing the most pronounced effect (Figure 6I–M). This is consistent with their sensitive phenotype to submergence. Subsequently, we examined antioxidant enzyme gene transcript levels via qRT-PCR, and gene expression analysis further revealed the transcriptional basis for this synergistic effect. Co-overexpression of *CsATG8f* and *CsRAP2.12* produced a significant synergistic enhancement on the transcriptional levels of key antioxidant enzymes *CsSOD*, *CsPOD*, and *CsCAT* (Figure 6F–H), whereas their co-silenced exhibited the most pronounced transcriptional suppression (Figure 6N–P). As reported by other researchers, ROS accumulation in wheat roots under hypoxia stress induces autophagy to enhance cell survival [103]. *AtRAP2.12* both induces Hypoxia Responsive Universal Stress Protein 1 (HRU1) to regulate ROS levels and mediates RbohD-dependent ROS signaling to maintain H_2_O_2_ homeostasis in Arabidopsis [104]. The waterlogging tolerant sesame genotype EC377024 typically exhibits stronger antioxidant capacity and more active stress-response gene expression compared to the susceptible genotype IC129289 [105]. Based on these findings, we propose that the CsATG8f–CsRAP2.12 interaction may functionally upregulate the antioxidant system at the transcriptional or post-transcriptional level, thereby enhancing submergence tolerance.

### 3.4. CsATG8f and CsRAP2.12 Can Induce Elevated Endogenous GABA Levels to Enhance Tea Plants’ Tolerance to Submergence

GABA plays multiple roles in plant responses to a range of abiotic stresses, including drought, salinity, waterlogging, and hypoxia, by regulating carbon-nitrogen balance, osmoregulation, antioxidant stress, and maintaining cytoplasmic pH homeostasis [106,107]. In rice coleoptiles, anaerobic conversion of inorganic nitrogen to alanine and GABA/glutamate under hypoxia may contribute to replenishing ethanol fermentation to sustain glycolytic energy production [108]. Hypoxia-induced GABA accumulation is most pronounced, a phenomenon reported across numerous plant species [109,110,111,112,113]. In this study, under submergence, GABA and Glu contents were significantly elevated in target gene-silenced plants exhibiting submergence-sensitive phenotypes compared to control (Figure 7M,N). Conversely, in target gene overexpression experiments, Glu levels in overexpression lines showed no difference from control, while GABA content was markedly reduced (Figure 7F,G). Gene expression analysis further revealed that, compared to control, GABA synthesis-related genes (*GAD1/2/3*, *AMADH*) were significantly downregulated in the overexpression lines under submergence, while GABA degradation-related gene *GABA-T2* showed significant upregulation (Figure 7A–E). In the silenced lines, GABA synthesis-related genes showed varying degrees of upregulation compared to the control, while *GABA-T2* expression was significantly downregulated (Figure 7H–L). These findings indicate that tea plants attempt to mitigate stress damage by increasing GABA content. This observation aligns with previous studies. *MdATG18a* overexpression enhances alkaline tolerance and GABA shunting in apple [114]. GABA may play a crucial role in plant adaptation to intense light and heat stress by promoting autophagy [115]. In Sindora glabra, *SgATG8a* is a key protein regulating GABA-mediated terpenoid production and drought tolerance [116]. Collectively, these pieces of evidence suggest that GABA-autophagy crosstalk represents a conserved mechanism in plant stress adaptation. Integrating our findings, submergence (hypoxia stress) significantly induces GABA accumulation, indicating GABA may contribute to plant adaptation to submergence by promoting autophagy.

### 3.5. Limitations and Future Perspectives

It is necessary to acknowledge the limitations of this study. Our experiments were conducted under controlled laboratory conditions, which facilitated the precise isolation and analysis of hypoxia stress and its signaling pathways. However, the natural environment of tea plants is far more complex, involving dynamic interactions between multiple abiotic stresses (such as light, temperature, and soil moisture) and concurrent biotic stresses. These interacting variables may modulate the hypoxia response mechanisms described herein, leading to distinct phenotypic outcomes or adaptive strategies under field conditions. Consequently, the findings of this study provide only a foundational mechanistic framework, requiring further validation under more realistic, multi-stress conditions. Future research could focus on: (1) validating key molecular components identified in this study (e.g., *CsATG8s*, ERF-VII hypoxia response transcription factors, GABA shunt genes) in field tea plants subjected to natural waterlogging stress; (2) investigating crosstalk between hypoxia signaling and other stress response pathways (e.g., drought, salinity, pathogen infection) to gain a more comprehensive understanding of plant resilience in complex environments.

## 4. Methods and Materials

### 4.1. Plant Materials, Growth Environment, and Stress Treatments

The experiment utilized one-year-old ‘Zhongcha 108’ tea seedlings as experimental material, all sourced from Nanjing Yarun Tea Industry Co., Ltd. (Nanjing, China). As an artificially bred variety with a well-documented breeding history, ‘Zhongcha 108’ facilitates genetic and molecular breeding research. The seedlings were cultivated in a mixture of nutrient soil:vermiculite:perlite at a 2:1:1 ratio under a 16 h/8 h photoperiod and a day/night temperature of 25 °C/22 °C. After one week of acclimatization, healthy seedlings with consistent growth were selected for treatment.

Vacuum treatment: Tea seedlings were placed in food-grade vacuum bags. Using a vacuum sealer (MS1160, Magic Seal, Yanguan, China), air was extracted for approximately 30 s, removing as much air as possible without damaging the seedlings before sealing. Treatment continued under the same photo period (16 h/8 h) and temperature conditions (day/night: 25 °C/22 °C). Samples were collected at 0 h, 3 h, 6 h, 12 h, and 24 h, consisting of the first and second leaves of each seedling. Samples were immediately immersed in liquid nitrogen for rapid freezing and stored at −80 °C for later use. Each treatment group consisted of 5–10 seedlings per genotype.

The submergence treatment was adapted from the method described by Liang Chen et al. with minor modifications [77]. Entire one-year-old tea seedlings, including soil, were completely submerged in plastic hydroponic containers with water levels maintained 5–10 cm above the plant apex. Plants were placed under a 16 h/8 h photoperiod with a day/night temperature of 25 °C/22 °C. Phenotypes were recorded or plant samples collected at designated time points. Each genotype comprised 5–10 tea seedlings.

Each biological replicate consisted of a pooled sample obtained from 5 to 10 uniformly growing tea plant young leaves per treatment group. Tissues from these plants were combined immediately snap-frozen in liquid nitrogen, and ground into fine powder for subsequent extraction of total RNA, proteins, or metabolites. All analyses (qRT-PCR, GABA assay, antioxidant enzyme assays) were performed using these biological replicate samples.

### 4.2. RNA Extraction and Real-Time Quantitative PCR

RNA extraction was performed according to the SteadyPure Plant RNA Quick Extraction Kit (Accurate Biology, AG21040, Nanjing, China) protocol. RNA concentration and quality were measured using a spectrophotometer (Nano-300, Allsheng, Hangzhou, China), with the elution buffer from the extraction kit serving as a blank control. cDNA was obtained using reverse transcription reagents (Accurate Biology, AG11728, Nanjing, China). Quantitative primers were designed using Primer Premier 5.0 software, with sequences listed in Table S1. Primer specificity was verified via conventional PCR reaction followed by gel electrophoresis. A single band indicated primer specificity and suitability for subsequent experiments. qRT-PCR was performed using a quantitative real-time PCR system (CFX96, Bio-Rad, Hercules, CA, USA) with the following reaction program: pre-denaturation at 95 °C for 30 s; annealing at 60 °C for 15 s; extension at 72 °C for 15 s; repeated 40 cycles. β-actin served as the internal control gene, and gene expression levels were calculated using the 2^−∆∆Ct^ method.

### 4.3. Subcellular Localization

Using homologous recombination, the full-length *CsATG8f* CDS was cloned into the pBI121-GFP expression vector to generate the pBI121-GFP-*CsATG8f* construct. The full-length *CsRAP2.12* CDS was cloned into the pBI121-GFP vector to generate the pBI121-GFP-*CsRAP2.12* construct. The constructs pBI121-GFP-*CsATG8f*, pBI121-GFP-*CsRAP2.12*, and the pBI121-GFP control were separately transformed into Agrobacterium GV3101. The transformed bacteria were then individually injected into tobacco (*Nicotiana tabacum*) leaves, kept in the dark for 24 h followed by 48 h under normal growth conditions. Leaf sections near the injection sites were prepared into plant slides and examined for GFP fluorescence using confocal microscope (LSM800, Zeiss, Oberkochen, Germany).

### 4.4. Chlorophyll In Vivo Fluorescence

Photographs of phenotypes and chlorophyll fluorescence images of tea plants with different genotypes were captured at 0h and 24h after submergence treatment. Chlorophyll fluorescence phenotypes were measured and recorded using a modulated chlorophyll fluorescence in vivo imaging system (PlantView 230F, Biolight Biotechnology Co., Ltd., Guangzhou, China). Prior to measurement, leaves were dark-adapted for 30 min to fully open the PSII reaction center. Maximum photochemical quantum yield (Fv/Fm) was determined and calculated using the instrument’s built in saturation pulse light source.

### 4.5. MDC Staining

Monodansylcadaverine (MDC) is an acidophilic fluorescent dye that specifically binds to lipid components (such as phosphatidylethanolamine) in autophagosome membranes. Under fluorescence microscopy, it selectively labels autophagosomes and emits green fluorescence. Fresh root tips of treated tea seedlings, approximately 1 cm in length, were cut and immersed in a 100 μM MDC solution (Sigma-Aldrich, 30432, St. Louis, MO, USA). The samples were stained under vacuum and in the dark for 30 min. Subsequently, they were thoroughly rinsed with 0.01M PBS (1×, pH 7.2–7.4, Solarbio, P1020, Beijing, China). Observation was performed using an LSM 800 confocal microscope with excitation at 405 nm and emission at 450–570 nm. To assess autophagy activity, at least 6–10 images per treatment were quantified to evaluate autophagic structures.

### 4.6. Agrobacterium-Mediated Transient Overexpression in Tea Seedlings

Healthy tea seedlings with similar growth vigor were infiltrated with Agrobacterium tumefaciens suspensions carrying either the target gene fragment plasmid or the pBI121-GFP plasmid (control). After incubated in the dark for 24 h and then under a light cycle for another 24 h, submergence treatment was initiated. Samples were collected after 24 h of treatment and stored at −80 °C. Gene expression levels were detected via qRT-PCR, and overexpression plants were selected for subsequent experiments.

### 4.7. Tobacco Rattle Virus (TRV)-Induced Gene Silencing (VIGS)

The insert fragments were generated using the online analysis website “https://vigs.solgenomics.net/ (accessed on 3 March 2025)”. The gene PCR amplification specific primers are listed in Table S1. The amplified fragments were ligated into the TRV2 vector to obtain the TRV2-*csatg8f* and TRV2-*csrap2.12* constructs, which were then transformed into Agrobacterium GV3101 strain. Infection methods and treatment conditions were consistent with those used in the overexpression experiments. Samples were collected at designated time points and flash-frozen. Gene expression levels were detected via qRT-PCR, and silenced plants were selected for subsequent experiments.

### 4.8. Physiological Parameter Measurement

Malondialdehyde (MDA) content was determined using the Malondialdehyde (MDA) Content Assay Kit (BC6415, Solarbio, Beijing, China). Hydrogen peroxide (H_2_O_2_) content was measured using the Hydrogen Peroxide assay kit (A064-1-1). Superoxide dismutase (SOD) activity was assessed using the Superoxide Dismutase (SOD) assay kit (A001-3-2). Catalase (CAT) activity was measured using the Catalase (CAT) assay kit (A007-1-1). Peroxidase (POD) activity was determined using the Peroxidase assay kit (A084-3-1). All enzyme assay kits were purchased from Nanjing Jiancheng Bioengineering Institute (Nanjing, China).

### 4.9. γ-Aminobutyric Acid Content Determination

Weigh 0.2 g of liquid nitrogen-ground sample into a 10 mL centrifuge tube. Add 2 mL of 0.02 mol/L HCl, shake to mix thoroughly, and extract at 4 °C for 8 h. Centrifuge the mixture at 15,000 rpm for 15 min. After centrifugation, transfer 2 mL of the supernatant to a new centrifuge tube. Then add an equal volume (2 mL) of 4% sulfosalicylic acid solution and mix thoroughly. Prior to transferring the sample to the injection vial, filter it through a 0.22 μm organic-based filter membrane. Analysis was performed using an amino acid analyzer (Hitachi L-8900, Tokyo, Japan).

### 4.10. Y2H Assay

The full-length CDS of *CsRAP2.12* was cloned into the pGADT7 vector to obtain pGADT7-*CsRAP2.12*. Simultaneously, the full-length CDS of *CsATG8f* was amplified using primers and inserted into the pGBKT7 vector to yield the pGBKT7-*CsATG8f* construct. The pGADT7-*CsRAP2.12* and pGBKT7-*CsATG8f* constructs were co-transformed into the yeast strain Y2H Gold. The transformed yeast cells were plated on TDO medium (SD/-Trp-Leu) and incubated at 28 °C for 2–3 days. Single colonies were then transferred to QDO medium (SD/-Trp-Leu-His-Ade) with or without X-α-gal for interaction screening. Prior to the experiment, it was necessary to confirm whether pGBKT7-CsATG8f exhibited self-activation.

### 4.11. BiFC Assay

The *CsATG8f* CDS was cloned into the pCAMBIA1300-cYFP vector to generate the pCAMBIA1300-cYFP-*CsATG8f* construct. Similarly, the *CsRAP2.12* CDS was used to generate the pCAMBIA1300-nYFP-*CsRAP2.12* construct. Agrobacterium tumefaciens strain GV3101 harboring both nYFP and cYFP derivative constructs was co-infiltrated into tobacco leaves. After 72 h of cultivation, YFP fluorescence signals were observed using an LSM 800 confocal microscope (Zeiss, Oberkochen, Germany).

### 4.12. LCI Assay

The full-length CDS of *CsATG8f* and *CsRAP2.12* were, respectively, cloned into the N-terminal and C-terminal regions of the LUC reporter gene, generating the nLUC-*CsATG8f* and cLUC-*CsRAP2.12* constructs. The constructed expression vectors were transformed into Agrobacterium GV3101 and then infiltrated into tobacco leaves. LUC activity was imaged and analyzed using in vivo plant imaging (PIXIS 1024B, Princeton Instruments, Trenton, NJ, USA) after 48–72 h of cultivation.

### 4.13. Data Analysis

All experiments were performed with at least three biological replicates. Data analysis was conducted using SPSS 20.0. Significance across multiple groups was determined using one-way ANOVA with Duncan’s multiple range test. GraphPad Prism 9.5 was used for data visualization.

## 5. Conclusions

**Figure 8:**
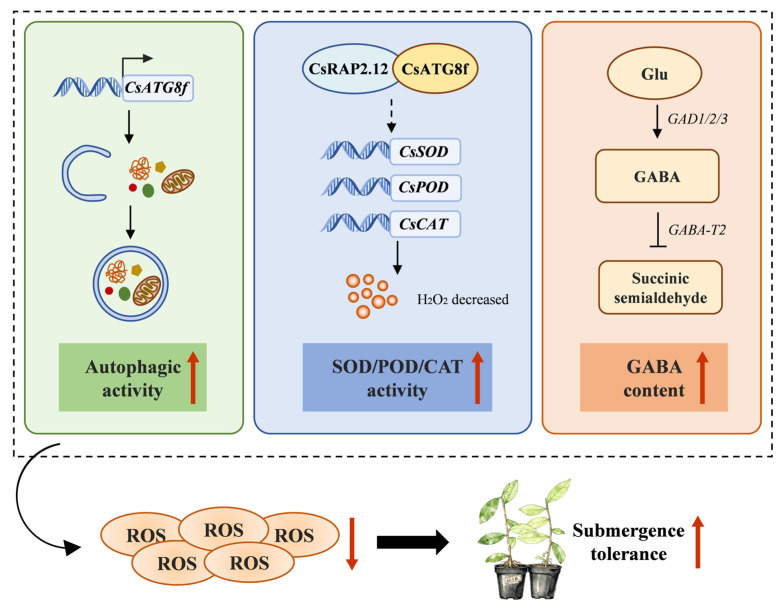
A proposed regulatory model for the roles of *CsATG8f* and *CsRAP2.12* in conferring submergence resistance in tea plants. Overexpression of *CsATG8f* enhances autophagic activity in tea plants under submergence. The interaction between CsATG8f and CsRAP2.12 may promote tea plants’ tolerance to submergence by promoting oxidative stress responses through an atypical fermentation pathway. Endogenous GABA levels increase in tea plants in response to submergence. Solid arrow indicates promotion. T-bar indicates inhibition. Dotted arrow indicates a hypothesized interaction or regulatory relationship.

In conclusion, we investigated the role of *CsATG8f* in mediating autophagy and regulating tolerance to submergence (hypoxia stress) in tea plants and proposed a regulatory model (Figure 8). Our findings indicate that overexpression of *CsATG8f* significantly increases the number of autophagosomes in the roots of tea plants under submergence. We also identified a physical interaction between the hypoxia-responsive transcription factors *CsRAP2.12* and *CsATG8f*. Integrating these findings, we hypothesize that the CsATG8f-CsRAP2.12 complex suppresses expression of downstream hypoxia-response genes to mitigate ethanol toxicity and energy depletion, thereby enhancing tea plant tolerance to submergence. This suggests a potential mechanism whereby the CsATG8f-CsRAP2.12 module may optimize adaptive responses by promoting autophagy-mediated antioxidant systems rather than solely activating classical fermentation pathways. Additionally, we observed that tea plants sensitive to submergence activate endogenous GABA levels to mitigate damage caused by submergence. However, as a complex abiotic stress involving hypoxic and energy-depleted responses, the regulatory network of submergence is intricate and multifaceted. The molecular mechanisms underlying these processes warrant further investigation. This study enhances our understanding of autophagy’s role in promoting tea plants’ adaptation to submergence. These findings provide valuable insights for future tea plant breeding efforts.

## Data Availability

The original contributions presented in this study are included in the article/Supplementary Material. Further inquiries can be directed to the corresponding author.

## Supplementary Materials

The following supporting information can be downloaded at: https://www.mdpi.com/article/10.3390/ijms27010235/s1.
