# Supplementary material for: Interaction Between CsATG8f and CsRAP2.12 Modulates Antioxidant Defense and Hypoxia Response During Submergence in Camellia sinensis
Source: Int J Mol Sci. 2025 Dec 25;27(1):235. doi: 10.3390/ijms27010235 (PMC12785919; doi:10.3390/ijms27010235)
Supplement: Supplementary file 1 [file ijms-27-00235-s001.zip › Supplemental Figures.pdf]

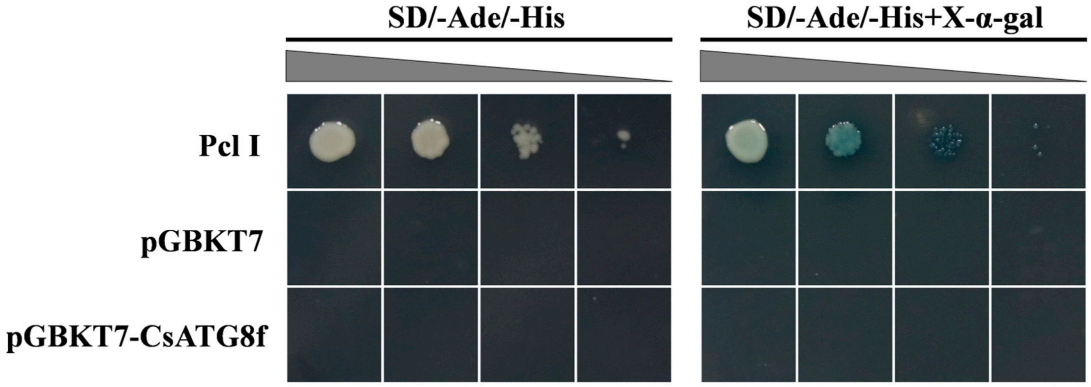

Figure S1. *CsATG8f* autoactivation verification. Results showed that *CsATG8f*-transformed yeast cells failed to grow on selective medium (SD/-Ade-His+X- α -gal).

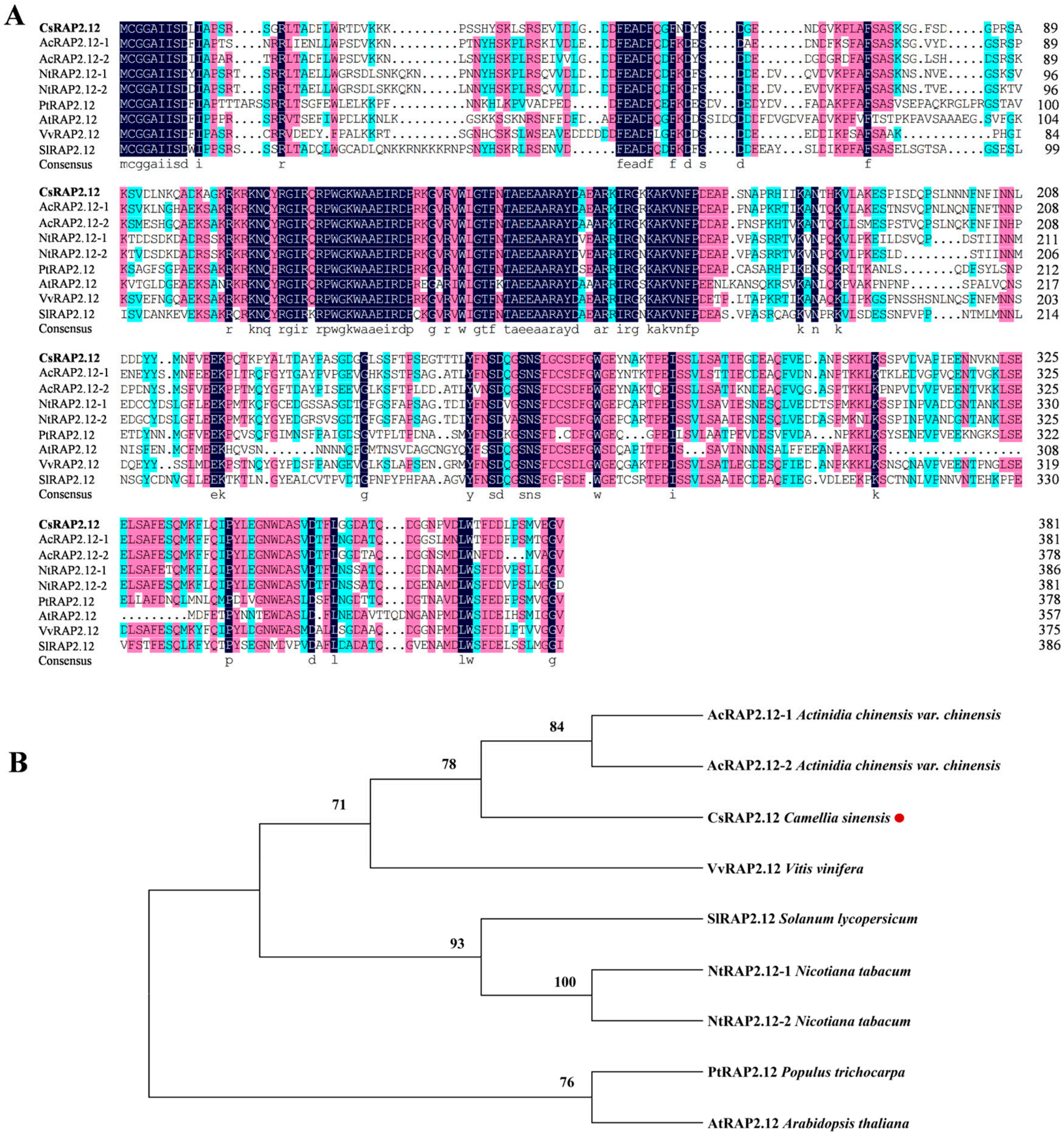

Figure S2. Sequence analysis of *CsRAP2.12*. (A) Protein domain alignment of RAP2.12 from different species. (B) Phylogenetic analysis of RAP2.12 from different species.

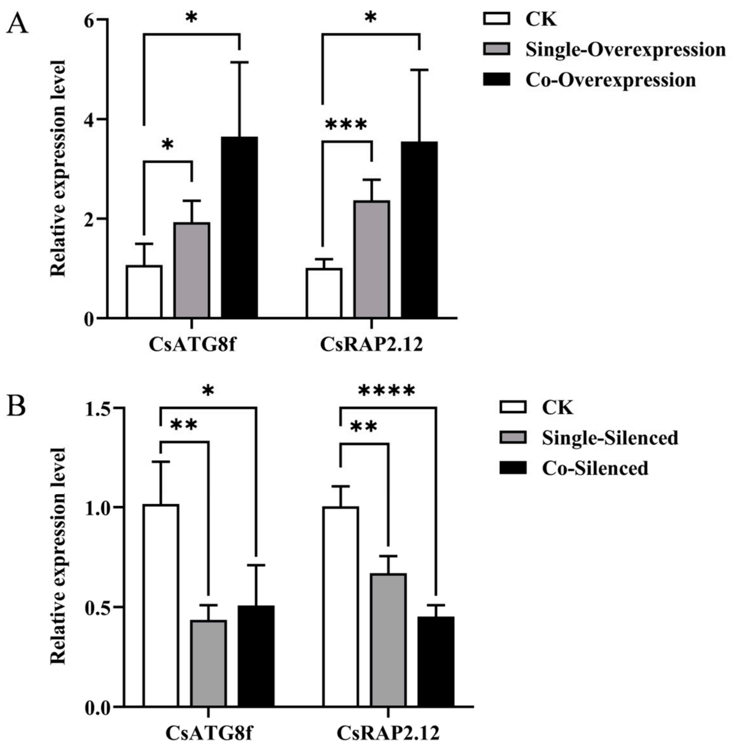

Figure S3. Efficacy of transient overexpression or transient silencing of target genes. (A) Overexpression efficiency of *CsATG8f* and *CsRAP2.12* in transient overexpression assay. (B) Silencing efficiency of *CsATG8f* and *CsRAP2.12* in transient silencing assay. Data are expressed as mean ± SD (n = 3). Statistically significant differences between groups are indicated by asterisks (*$p < 0.05$, **$p < 0.01$, ***$p < 0.001$, ****$p < 0.0001$, one-way ANOVA).
